# TTN/OBSCN ‘Double‐Hit’ predicts favourable prognosis, ‘immune‐hot’ subtype and potentially better immunotherapeutic efficacy in colorectal cancer

**DOI:** 10.1111/jcmm.16393

**Published:** 2021-02-23

**Authors:** Zaoqu Liu, Libo Wang, Chunguang Guo, Long Liu, Dechao Jiao, Zhenqiang Sun, Kunpeng Wu, Yanan Zhao, Xinwei Han

**Affiliations:** ^1^ Department of Interventional Radiology The First Affiliated Hospital of Zhengzhou University Zhengzhou China; ^2^ Interventional Institute of Zhengzhou University Zhengzhou China; ^3^ Interventional Treatment and Clinical Research Center of Henan Province Zhengzhou China; ^4^ Department of Hepatobiliary and Pancreatic Surgery The First Affiliated Hospital of Zhengzhou University Zhengzhou China; ^5^ Department of Endovascular Surgery The First Affiliated Hospital of Zhengzhou University Zhengzhou China; ^6^ Department of Colorectal Surgery The First Affiliated Hospital of Zhengzhou University Zhengzhou China

**Keywords:** colorectal cancer, immunotherapy, mutation, OBSCN, TTN

## Abstract

Colorectal cancer (CRC) remains a leading cause of cancer‐related deaths worldwide. Although treatment strategies for solid tumours have been revolutionized by immunotherapy, only a small subset of CRC patients benefit. Using two‐independent cohorts, we found the common frequently mutated genes *TTN* and *OBSCN* had the significant correlation with higher tumour mutation burden (TMB) and favourable overall survival. *TTN* and *OBSCN* also displayed significant commutation phenomenon. Therefore, based on the status of *TTN* and *OBSCN*, we stratified patients into ‘Double‐WT’ phenotype, ‘Single‐Hit’ phenotype and ‘Double‐Hit’ phenotype. Importantly, the ‘Double‐Hit’ phenotype had favourable prognosis, low malignant events propensity, and highest TMB, immune cells infiltration abundance, *POLE* mutation rate, microsatellite instability ratio, as well as immune checkpoints expression compared with the other two phenotypes. These results indicated that the ‘Double‐Hit’ phenotype suggested ‘immune‐hot’ tumours and potentially better immunotherapeutic efficacy. Bioinformatic algorithm assessment of immunotherapy responses also confirmed this conclusion, and the ‘Double‐Hit’ phenotype was found to be a better predictor of immunotherapy than *PD‐L1*, *PD‐1*, *CTLA‐4*, TMB and microsatellite status. This study revealed CRC patients with TTN/OBSCN ‘Double‐Hit’ was significantly associated favourable prognosis, ‘immune‐hot’ subtype and potentially better immunotherapeutic efficacy.

## 1 INTRODUCTION

Colorectal cancer (CRC) is the fourth most prevalent cancer and the third most causes of cancer‐related mortality worldwide.^1^ Currently, treatment modalities for CRC include surgical resection, targeted therapy, adjuvant chemotherapy and radiotherapy. Nevertheless, thirty per cent of patients with CRC still develop recurrence or metastasis after first‐line therapy.^2^ Hence, the development of more effective treatments for CRC is an urgent need to improve clinical outcome.

Over the past decade, immunotherapy has illustrated tremendous sensation owing to its remarkable efficacy in the treatment of solid tumours.^3^ Immune checkpoint inhibitors (ICIs) facilitate the immune system to recognize and inhibit the essential targets on tumour cells such as *PD‐L1*, *PD‐1* and *CTLA‐4*.^4^ In CRC, immunotherapy was approved in 2017 for the therapy of patients with DNA mismatch repair deficient (dMMR) or advanced microsatellite instability (MSI). Apart from MSI, there are other classification systems to stratify patients, such as consensus molecular subtypes, tumour mutation burden (TMB), *PD‐1*/*PD‐L1, CTLA‐4* and tumour immune microenvironment.^3^ Nevertheless, these classification methods are not perfectly predicting the responses to immunotherapy, and only a small subset of CRC patients benefit.^5^ In view of the serious adverse reactions and high cost of immunotherapy, it is necessary to explore new biomarkers for effective immunotherapy management in CRC patients.

Genetic mutations in CRC were also assessment indicators for immunotherapy.^6^ For example, patients with *POLE* mutation generally benefit more from ICIs therapy. TMB has also been reported to be significantly associated with the immunological response and treatment of cancer. The mutation landscape of CRC has been well demonstrated worldwide. The Cancer Genome Atlas (TCGA) and the International Cancer Genome Consortium (ICGC) have described large‐scale comprehensive mutational characterization of CRC. Substantial efforts have been put into identifying driven genes such as *APC*, *TP53*, *PIK3CA*, *KRAS*, *SMAD4* and *BRAF*. Mutations in these genes are involved in the initiation, progression, treatment, drug resistance, prognosis and recurrence of CRC.^7^, ^8^, ^9^, ^10^ Therefore, we speculate that there are some latent frequently mutated genes (FMGs) also could identify patients who might benefit form immunotherapy. Compared with traditional immunotherapy biomarkers (*PD‐1*/*PD‐L1*, *CTLA‐4* and TMB), binary genetic mutation clinical data do not require defining cut‐off values to stratify patients, which is more convenient for clinical management and translation.

In the present research, we firstly demonstrated that mutations in *TTN* and *OBSCN* were significantly related to TMB and overall survival (OS) in patients with CRC *TTN* and *OBSCN* also displayed significant commutation phenomenon. Therefore, according to the status of *TTN* and *OBSCN*, we stratified patients into ‘Double‐WT’ phenotype, ‘Single‐Hit’ phenotype and ‘Double‐Hit’ phenotype. The three phenotypes displayed substantial differences in prognosis, malignant events propensity, molecular characteristics and immune landscape, which indicated that each phenotype should require a specific therapeutic strategy. Notably, the ‘Double‐Hit’ phenotype displayed the highest TMB, MSI, immune cells infiltration, ICPs expression, *POLE* mutation rate and immunotherapy response rate, suggesting ‘immune‐hot’ tumours. These results indicated the ‘Double‐Hit’ phenotype might be a reliable biomarker for predicting immunotherapy responses. Moreover, we identified potential chemotherapeutic agents with specific sensitivity among three phenotypes, which provided a resource for precision chemotherapy in the *TTN*/*OBSCN* mutant phenotypes.

## 2 MATERIALS AND METHODS

### 2.1 Data sources and processing

Somatic gene mutations data of American CRC patients (n = 535) and Chinese CRC patients (n = 318) were enrolled from TCGA (http://portal.gdc.cancer.gov/) and ICGC (http://dcc.icgc.org/) databases, respectively. The RNA‐seq data (FPKM normalized) of TCGA‐CRC cohort were derived from the UCSC‐Xena database and were further transformed to log_2_ (TPM + 1). CRC patients were removed if they lacked somatic mutations, RNA‐seq, survival data and received neo‐adjuvant therapy.

### 2.2 Estimate TMB in terms of per megabase

Tumour mutation burden was measured as the number of coding, somatic, base substitution and indels mutations per megabase (MB) of the targeted territory. We counted all base substitutions and indels in the coding region of targeted genes. Synonymous mutations that do not result in amino acid variations are not counted. Using the ‘tmb’ function in ‘maftools’ R package, we calculated the TMB for each patient.^11^

### 2.3 Functional analysis and immune cells infiltration assessment

To further analyse the latent functions underlying the *TTN*/*ONSCN* ‘Double‐Hit’ phenotype, the gene‐set enrichment analysis (GSEA) algorithm was carried out to identify enriched dramatically terms correlated with Kyoto Encyclopedia of Genes and Genomes (KEGG) pathway and biological process of gene ontology (GO). We set the number of random permutations as 1000 to generate a normalized enrichment score (NES) and false discovery rate (FDR) *q* value. We deemed the terms with NES >2 and FDR *q* value <0.001 were strikingly enriched. Subsequently, the single‐sample gene‐set enrichment analysis (ssGSEA) approach was utilized to evaluate the infiltration abundance of 28 immune cells in the tumour microenvironment (TME). We retrieved the gene sets for annotating each TME cell from the study of Charoentong, which contained miscellaneous innate and adaptive immune cells such as CD4/CD8^+^ T cell, B cell, natural killer T cell, macrophage, dendritic cell and monocyte.^12^

### 2.4 Bioinformatic assessment of immunotherapy

The Subclass Mapping (SubMap) algorithm is an unsupervised clustering approach, which reveals common subtypes between independent cohorts. Using SubMap method, we measured the similarity pattern of mRNA expression between the three subtypes and the patients with different immunotherapy responses.^13^ An FDR for the two subclasses less than 0.05 indicated a dramatical similarity. Subsequently, we used the Tumor Immune Dysfunction and Exclusion (TIDE) web tool (http://tide.dfci.harvard.edu/) to predict ICIs clinical response based on the pre‐treatment expression data of tumours.^14^ The TIDE module assesses immune evasion by integrating the expression signatures of T cell dysfunction and T cell exclusion. Using the TIDE framework, we obtained the outcome of the bioinformatic assessment of immunotherapy for each patient.

### 2.5 Chemotherapeutic response prediction

A previous work has proposed a ridge regression model to evaluate the imputed response to 138 chemotherapeutic agents based on pharmacogenomics and gene expression data.^15^ The ‘pRRophetic’ R package was applied to perform the prediction process. The half‐maximal inhibitory concentration (IC50) was utilized to quantified drug sensitivity, and the lower the IC50, the higher the sensitivity. In order to identify potential chemotherapeutic agents with specific sensitivity among three phenotypes through the following procedure: (a) The Kolmogorov‐Smirnov normality test indicated that the imputed drug response (IC50) belonged to be a skewed distribution (*P* < .05), thus, Kruskal‐Wallis and Wilcoxon rank‐sum tests were applied to compare the differences; (b) The *P*‐values were adjusted by Benjamini‐Hochberg (BH) multiple test correction, and an FDR <0.05 was considered significant; (c) For each potential chemotherapeutic agent, if the Kruskal‐Wallis test FDR < 0.05 and the IC50 of one subtype was predominantly lower than that of the other two subtypes (Wilcoxon rank‐sum test FDR <0.05), the subtype was thought to be more sensitive to the drug; (d) In terms of the median IC50 value of an antitumour drug with specific sensitivity in each phenotype, the sensitivity of three phenotypes was labelled ‘High sensitivity’, ‘Intermediate sensitivity’ and ‘Low sensitivity’ according to the magnitude of the median IC50 value.

### 2.6 Statistical analysis

The waterfall plot of FMGs was summarized and visualized with ‘maftools’ R package, and the Fisher exact test was applied to assess the co‐occurrence or mutually exclusive significance of FMGs. The Kolmogorov‐Smirnov normality test *P*‐value of TMB, immune cells infiltration abundance, immune checkpoints (ICPs) expression and IC50 were all less than 0.05. Therefore, the Wilcoxon rank‐sum and Kruskal‐Wallis tests were performed to compare the differences of two and multiple groups, respectively. Comparisons between categorical variables using Fisher's exact test or chi‐squared test. The ‘clusterProfiler’ R package was utilized to perform GSEA process.^16^ The Kaplan‐Meier analysis was performed via ‘survival’ R package, and the log‐rank test was applied to compare the survival differences among three phenotypes. FDR was generated via BH multiple test correction. All *P*‐values were two‐sided, and *P* < .05 was considered significant. All data processing, statistical analysis and plotting were performed in R 4.0.2 software.

## 3 RESULTS

### 3.1 Landscape of somatic mutations in CRC

**FIGURE 1:**
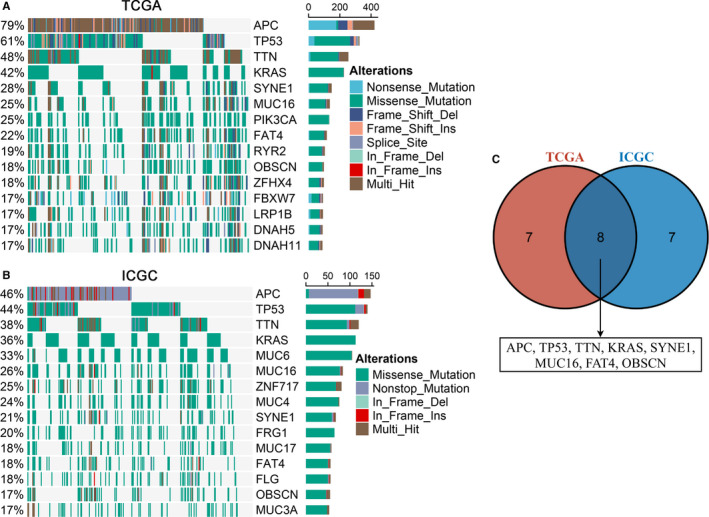
Landscapes of frequently mutated genes (FMGs) in CRC. A, B, Oncoplot depicts the FMGs of CRC in the TCGA (A) and ICGC (B) cohorts. The left panel shows mutation rate, and genes are ordered by their mutation frequencies. The right panel presents different mutation types. C, Venn diagram of FMGs covered by both TCGA and ICGC cohorts

A total of 15 FMGs were defined in American CRC patients from the TCGA cohort, including *APC* (79%), *TP53* (61%), *TTN* (48%), *KRAS* (42%), *SYNE1* (28%), *MUC16* (25%), *PIK3CA* (25%), *FAT4* (22%), *RYR2* (19%), *OBSCN* (18%), *ZFHX4* (18%), *FBXW7* (17%), *LRP1B* (17%), *DNAH5* (17%) and *DNAH11* (17%; Figure 1A). Meanwhile, we also defined 15 FMGs in Chinese CRC patients from the ICGC cohort, including *APC* (46%), *TP53* (44%), *TTN* (38%), *KRAS* (36%), and *MUC6* (33%), *MUC16* (26%), *ZNF717* (25%), *MUC4* (24%), *SYNE1* (21%), *FRG1* (20%), *MUC17* (18%), *FAT4* (18%), *FLG* (18%), *OBSCN* (17%) and *MUC3A* (17%; Figure 1B). Of note, some FMGs were shared in both American and Chinese patients, including *APC*, *TP53*, *TTN*, *KRAS*, *SYNE1*, *MUC16*, *FAT4* and *OBSCN* (Figure 1C). Therefore, we focused on these common FMGs in subsequent analysis.

### 3.2 *TTN* and *OBSCN* mutation associated with TMB and OS

**FIGURE 2:**
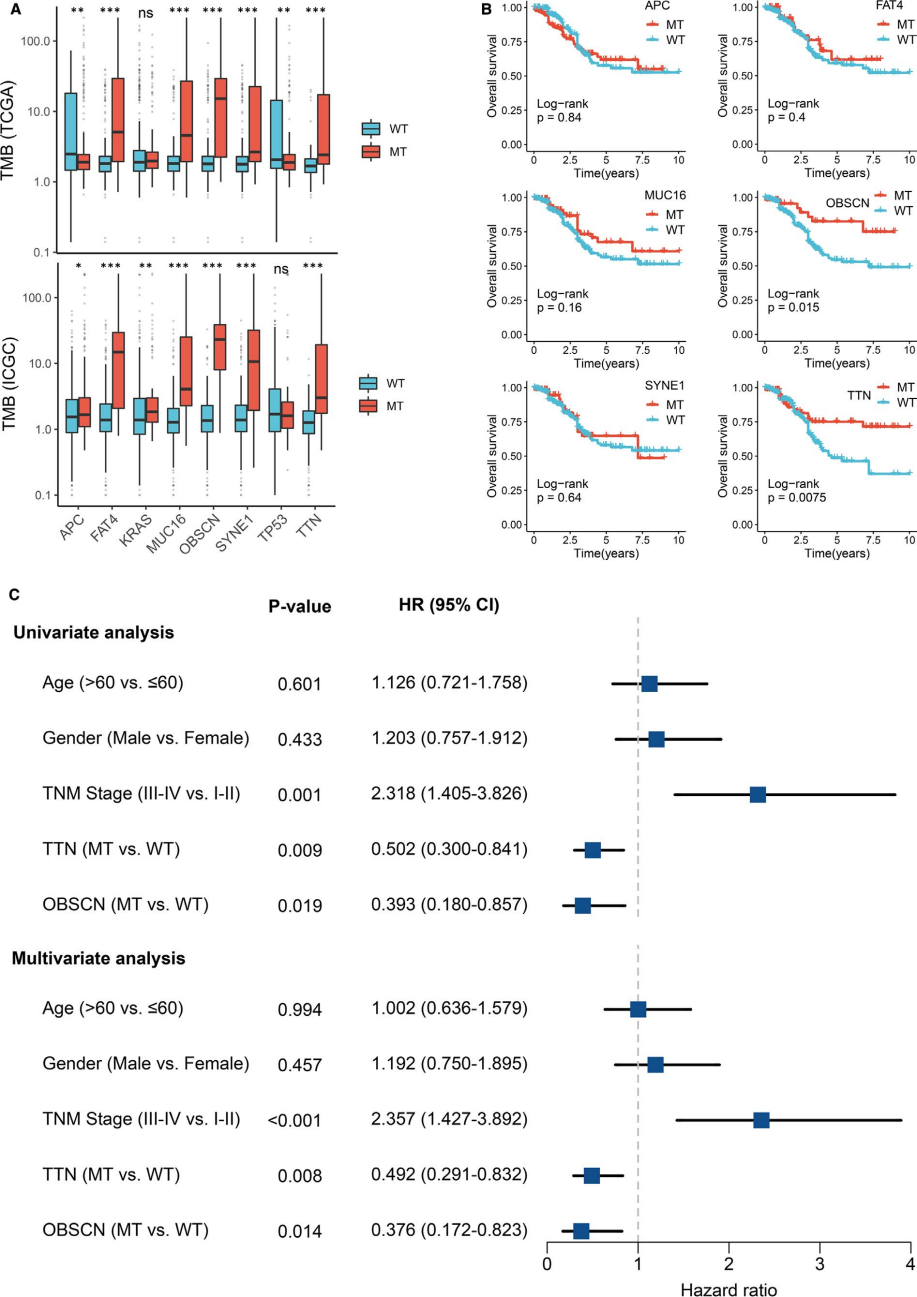
Gene mutations are associated with TMB and clinical prognosis. A, Most gene mutations are associated with a higher TMB. ns *P* > .05; *P < .05; **P < .01; ***P < .001. B, Kaplan‐Meier survival analysis of patients with gene mutations. C, Univariate and multivariate Cox regression analysis of TTN and OBSCN mutations. MT, mutant type; WT, wild‐type

The TMB in the TCGA cohort ranged from 0.04 to 214.34/MB with a median value of 1.82/MB, and the TMB in the ICGC cohort ranged from 0.01 to 231.46/MB with a median value of 1.51/MB. Among these common FMGs, patients with mutations in *APC*, *FAT4*, *MUC16*, *OBSCN*, *SYNE1* and *TTN* demonstrated significantly higher TMB in two cohorts (Figure 2A). Previous study has reported that the high‐TMB is dramatically associated with a favourable prognosis in CRC.^17^ Hence, Kaplan‐Meier survival analysis was further carried out to determine whether mutations in these FMGs related to higher TMB were also associated with OS in CRC patients. As illustrated in Figure 2B, patients with *TTN* or *OBSCN* mutations possessed a favourable OS (*P* < .05). Univariate Cox regression analysis displayed the hazard ratios (HRs) of *TTN* and *OBSCN* were 0.502 (95% confidence interval [CI]: 0.300‐0.841) and 0.393 (95% CI: 0.180‐0.857), respectively (*P* < .05; Figure 2C). Furthermore, multivariate analysis suggested that *TTN* and *OBSCN* mutations remained statistically significance (*P* < .05), and the HRs of *TTN* and *OBSCN* were 0.492 (95% CI: 0.291‐0.832) and 0.376 (95% CI: 0.172‐0.823), respectively (Figure 2C). This finding indicated that both *TTN* and *OBSCN* mutations were independent protective factors of OS in CRC.

### 3.3 *TTN/OBSCN* mutant phenotypes

**FIGURE 3:**
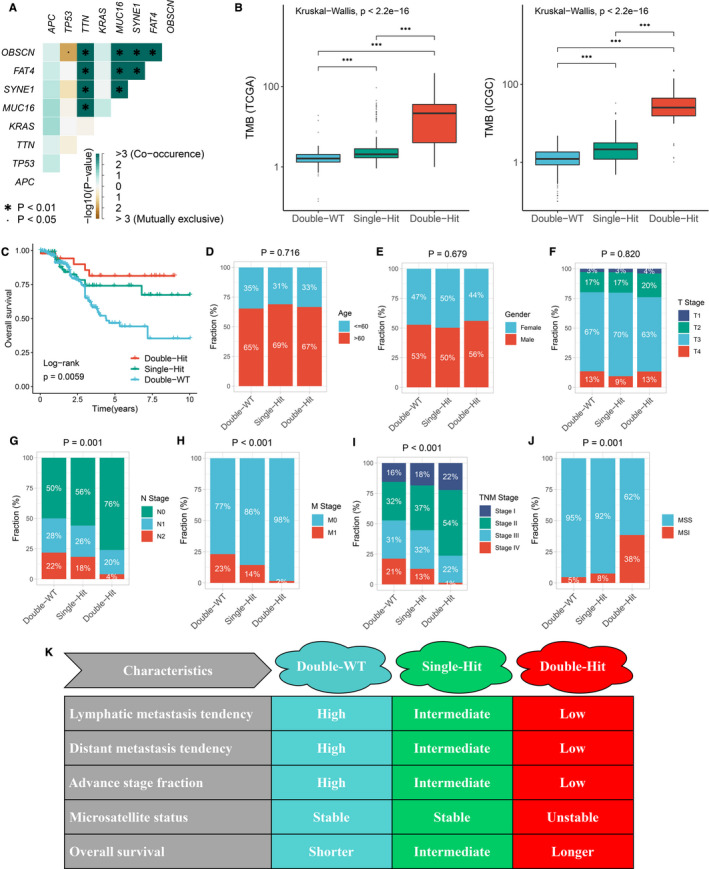
The difference of clinical characteristics among three TTN/OBSCN mutant phenotypes. A, Mutation co‐occurrence and exclusive relationships of eight FMGs. B, Distribution of TMB in the three phenotypes. ***P < .001. C, Kaplan‐Meier survival analysis of three phenotypes. D‐J, Composition percentage of age (D), gender (E), T stage (F), N stage (G), M stage (H), TNM stage (I) and microsatellite status among the three phenotypes (J). K, Summary table of clinical characteristics of the three phenotypes

We noted that *TTN* and *OBSCN* had predominant commutation phenomenon (*P* < .01; Figure 3A). Therefore, we suggested that the status of *TTN* and *OBSCN* might be tightly related to clinical outcome and underlying biological mechanisms of CRC patients. Subsequently, patients with the commutation of *TTN* and *OBSCN* were labelled ‘Double‐Hit’, patients with only one gene mutation (*TTN* or *OBSCN*) were labelled ‘Single‐Hit’, and patients with the double wild‐type of *TTN* and *OBSCN* were labelled ‘Double‐WT’. Interestingly, patients with ‘Double‐Hit’ phenotype had the highest TMB in TCGA and ICGC cohorts relative to the other two phenotypes (Figure 3B). Moreover, we observed that ‘Double‐WT’, ‘Single‐Hit’ and ‘Double‐Hit’ phenotypes suggested the shorter OS, intermediate OS and longer OS, respectively (*P* < .05; Figure 3C,K). The HR of the *TTN*/*OBSCN* mutation phenotypes was 0.568 (95% CI: 0.392‐0.824; *P* < .05; Figure S1). After controlling for age, gender and TNM stage, the *TTN*/*OBSCN* mutant phenotypes remained statistically significance (*P* < .05), which suggested that the *TTN*/*OBSCN* mutant phenotypes was also an independent protective factor of OS in CRC (Figure S1). There were no statistically significant differences in the age, sex or T stage of CRC patients among three phenotypes (Figure 3D‐F). In line with the prognosis results, patients with ‘Double‐WT’ tended to possess worse clinical outcome such as lymphatic metastasis, distant metastasis and advance stage, whereas patients with ‘Double‐Hit’ phenotype possessed lower propensity for malignancy events (*P* < .05; Figure 3G‐I,K). Notably, a large proportion of patients with MSI in ‘Double‐Hit’ phenotype, and most patients in the other two phenotypes had stable microsatellite status (*P* < .05; Figure 3J,K). Previous study has reported that MSI tumours had a more positive prognosis compared to microsatellite stable (MSS) tumours. Therefore, in aggregate, ‘Double‐Hit’ phenotype indicated favourable OS and lower propensity for malignancy events, ‘Double‐WT’ phenotype indicated adverse OS and higher propensity for malignancy events, whereas ‘Single‐Hit’ phenotype was intermediate between ‘Double‐Hit’ and ‘Double‐WT’ (Figure 3K).

### 3.4 *TTN/OBSCN* ‘Double‐Hit’ suggested ‘immune‐hot’ tumours

**FIGURE 4:**
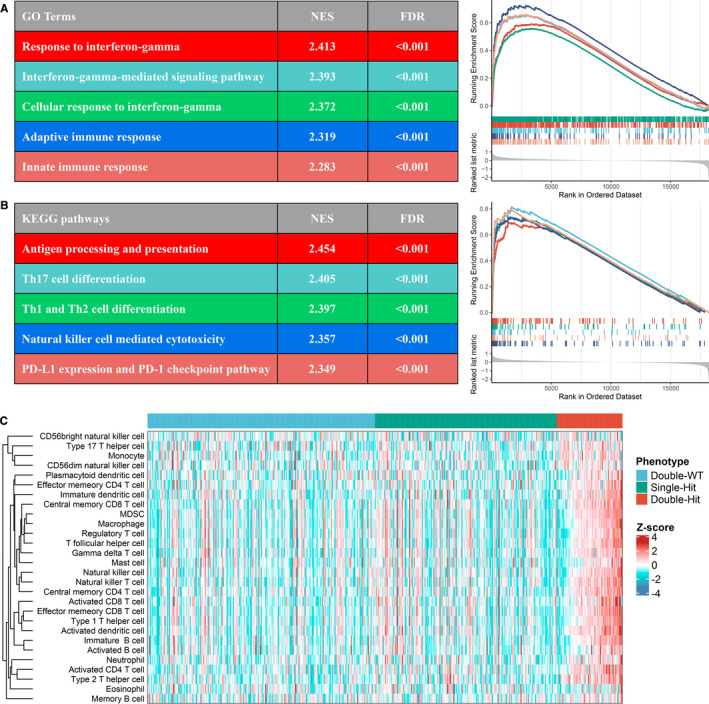
Functional and immune infiltration analysis. A, Top five GO terms enriched in the ‘Double‐Hit’ phenotype. B, Top five KEGG pathways enriched in the ‘Double‐Hit’ phenotype. C, Assessment of infiltration abundance of 28 immune cells in the three phenotypes

Based on the GSEA analysis, we observed a multitude of immune‐related GO terms were significantly enriched in ‘Double‐Hit’ phenotype, and the top five terms were ‘Response to interferon‐gamma’ (NES = 2.413, FDR < 0.001), ‘Interferon‐gamma‐mediated signalling pathway’ (NES = 2.393, FDR < 0.001), ‘Cellular response to interferon‐gamma’ (NES = 2.372, FDR < 0.001), ‘Adaptive immune response’ (NES = 2.319, FDR < 0.001) and ‘Innate immune response’ (NES = 2.283, FDR < 0.001; Figure 4A). The ‘Double‐Hit’ phenotype was also dramatically associated with abundant immune‐related KEGG pathways, and the top five pathways were ‘Antigen processing and presentation’ (NES = 2.454, FDR < 0.001), ‘Th17 cell differentiation’ (NES = 2.405, FDR < 0.001), ‘Th1 and Th2 cell differentiation’ (NES = 2.397, FDR < 0.001), ‘Natural killer cell mediated cytotoxicity’ (NES = 2.357, FDR < 0.001) and ‘PD‐L1 expression and PD‐1 checkpoint pathway’ (NES = 2.349, FDR < 0.001; Figure 4B). Moreover, the ssGSEA algorithm was utilized to further calculate the relative infiltration abundance of 28 immune cell types. In line with the above results, the infiltration abundance of most immune cells in ‘Double‐Hit’ phenotype was significantly higher than the other two phenotypes (*P* < .05), and ‘Single‐Hit’ phenotype was intermediate between ‘Double‐Hit’ and ‘Double‐WT’ (Figure 4C and Figure S2). Collectively, these findings indicated that *TTN*/*OBSCN* ‘Double‐Hit’ suggested ‘immune‐hot’ tumours, which has potential implications for optimizing immunotherapy.

### 3.5 Molecular characteristics and therapy implications of the *TTN*/*OBSCN* mutant phenotypes

**FIGURE 5:**
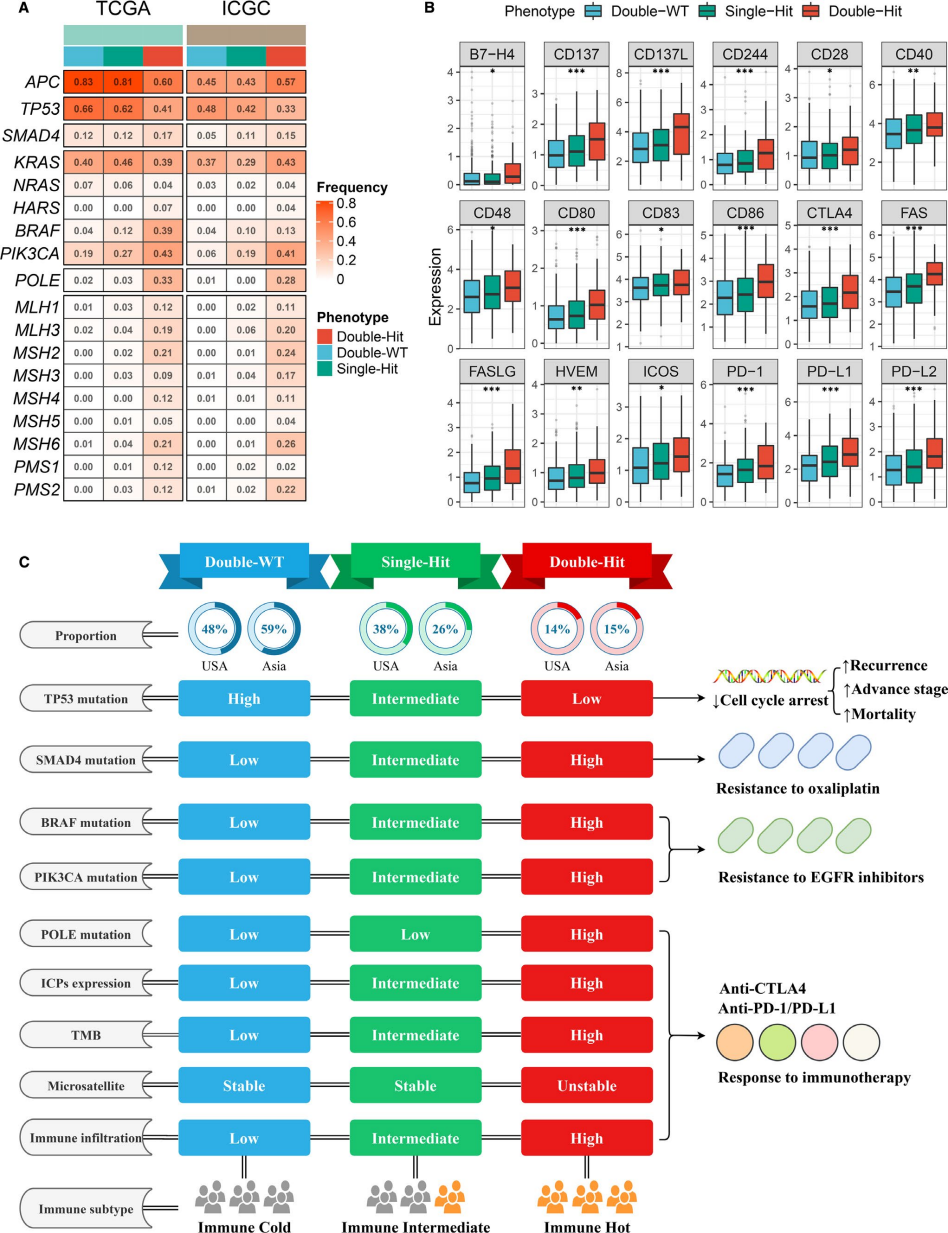
Molecular and immune landscape of three TTN/OBSCN mutant phenotypes. A, Mutation rate of driver genes among three TTN/OBSCN mutant phenotypes in TCGA and ICGC cohorts. B, Expression distribution of immune checkpoints in three phenotypes. *P < .05; **P < .01; ***P < .001. C, Summary of molecular and immune landscape of three phenotypes

Previous study has demonstrated that the mutations of some genes were predominantly associated with the initiation, progression and cancer therapy of CRC, such as *APC*, *TP53*, *SMAD4*, *RAS*, *BRAF*, P*IK3CA* and *POLE*.^18^ The inactive mutation of DNA mismatch repair (MMR) systems was reported to be dramatically correlated with MSI.^19^ We calculated the mutation rate of these genes in the three *TTN*/*OBSCN* mutant phenotypes (Figure 5A). Of note, the ‘Double‐Hit’ phenotype of American patients had the lowest *APC* mutation rate, whereas the ‘Double‐Hit’ phenotype of Chinese patients had the highest *APC* mutation rate (Figure 5A). Mutations in *TP53* were most significant in the ‘Double‐WT’ phenotype, which was associated with higher recurrence rate, advance stage and higher mortality in CRC (Figure 5A,C).^20^ There were no significant differences in the mutation frequency of RAS genes (including *KRAS*, *NRAS* and *HARS*) among the three phenotypes (Figure 5A). Mutations in *SMAD4* have been proven to increase resistance to oxaliplatin in CRC patients^21^ and were most common in ‘Double‐Hit’ phenotype (Figure 5A,C). Previous study has reported that patients with *BRAF* or *PIK3CA* mutations had superior resistance to *EGFR* inhibitors.^22^, ^23^ Thus, the ‘Double‐Hit’ phenotype with the highest rate of *BRAF* and *PIK3CA* mutations might benefit less from anti‐EGFR therapy.

The ‘Double‐Hit’ phenotype possessed the highest mutation rate of *POLE*, which was associated with longer disease‐free survival and better immunotherapy responses (Figure 5A).^24^ Almost all mutations in MMR systems occurred in the ‘Double‐Hit’ phenotype, which might explain the unstable microsatellite status of the ‘Double‐Hit’ phenotype (Figure 5A). Beyond that, the ‘Double‐Hit’ phenotype also had dramatically higher expression of ICPs, such as *PD‐1*, *PD‐L1*, *PD‐L2* and *CTLA‐4* compared with the other two phenotypes (Figure 5B,C). This provided more opportunity targets for ICIs therapy in the ‘Double‐Hit’ phenotype. Plus the previous results, patients with the ‘Double‐Hit’ phenotype displayed higher TMB, unstable microsatellite status and superior immune cells infiltration, suggesting an ‘immune‐hot’ subtype; patients with the ‘Single‐Hit’ phenotype displayed intermediate TMB, stable microsatellite status and intermediate immune cells infiltration, suggesting an ‘immune‐intermediate’ subtype; patients with the ‘Double‐WT’ phenotype displayed low‐TMB, stable microsatellite status and low immune cells infiltration, suggesting an ‘immune‐cold’ subtype (Figure 5C). Overall, the molecular characteristics of the *TTN*/*OBSCN* mutant phenotypes are of great significance for cancer therapy, particularly immunotherapy, and patients with the ‘Double‐Hit’ phenotype might benefit most from immunotherapy.

### 3.6 Assessment of immunotherapy and chemotherapy

**FIGURE 6:**
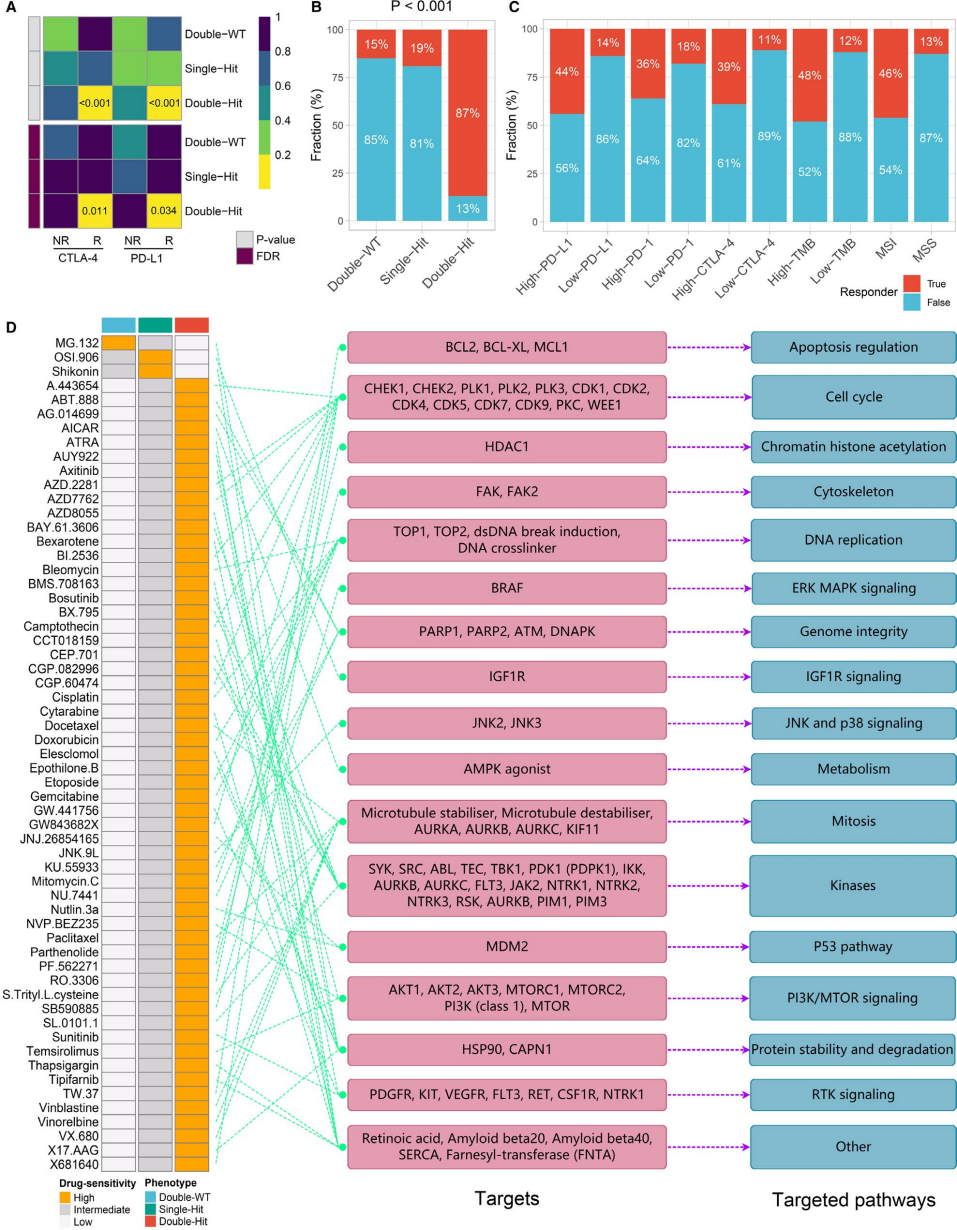
Assessment of immunotherapy and chemotherapy among three phenotypes. A, SubMap algorithm evaluated the expression similarity between the three phenotypes and the patients with different immunotherapy responses. B, Distribution of immunotherapy responders predicted by TIDE algorithm in three phenotypes. C, Distribution of immunotherapy responders in different groups. D, A total of 59 potential antitumour drugs with specific sensitivity to each phenotype were identified. The left panel is the drug names and the level of sensitivity in each phenotype, the middle panel is the drug‐targeted molecules, whereas the right panel represents the drug‐targeted pathways

We applied SubMap analysis to reveals common subtypes between independent cohorts and found significant expression similarity between the ‘Double‐Hit’ phenotype and patients with anti‐PD‐L1 and anti‐CTLA‐4 therapy (FDR < 0.05; Figure 6C). This confirms the above conclusion that immunotherapy was more effective in patients with ‘Double‐Hit’ phenotype than the other two phenotypes. The TIDE framework was further employed to evaluate the immunotherapy outcome of each patient, and we found the proportion of responders to immunotherapy with ‘Double‐Hit’ phenotype was the highest relative to the other two phenotypes (Double‐WT vs Single‐Hit vs Double‐Hit: 15% vs 19% vs 87%; *P* < .001; Figure 6B). In addition, CRC patients were divided into high‐PD‐L1 and low‐PD‐L1 or high‐PD‐1 and low‐PD‐1 or high‐CTLA‐4 and low‐CTLA‐4 or high‐TMB and low‐TMB or MSS and MSI groups by the median values of PD‐L1, PD‐1, CTLA‐4 and TMB or the status of microsatellite. In line with previous study, patients with high‐*PD‐L1*, *PD‐1*, *CTLA‐4*, TMB and MSI had more immunotherapy response rate compared with the other groups (Figure 6C).^3^ Notably, the proportion of responders with the ‘Double‐Hit’ phenotype was significantly higher than that of high‐PD‐L1, high‐PD‐1, high‐CTLA‐4, high‐TMB and MSI groups. These results suggested the *TTN*/*OBSCN* mutant phenotype might be a better predictor of immunotherapy than *PD‐L1*, *PD‐1*, *CTLA‐4*, TMB and microsatellite status.

Furthermore, using ‘pRRophetic’ R package, we evaluated the imputed responses to 138 chemotherapeutic agents in CRC patients to identify potential drugs with specific sensitivity to each phenotype.^15^ As displayed in Figure 6D, A total of 59 drugs were determined. For instance, MG‐132 targeting proteasome was more sensitive to patients with ‘Double‐WT’ phenotype; OSI‐906 targeting IGF1R signalling was more sensitive to patients with ‘Single‐Hit’ phenotype. Importantly, fifty‐six of these 59 drugs were specific sensitivity to patients with ‘Double‐Hit’ phenotype (Figure 6D). Of these 56 drugs, the majority of their targeted pathways were associated with tumour proliferation, such as cell cycle (seven drugs), DNA replication (seven drugs), mitosis (seven drugs), P53 pathway (two drugs) and PI3K/MTOR signalling (four drugs). Other pathways such as MAPK signalling (two drugs), kinases (five drugs), protein stability and degradation (five drugs) and RTK signalling (three drugs) were latent targets for patients with ‘Double‐Hit’ phenotype (Figure 6D). These results offered many opportunities for potential targeted therapies in the ‘Double‐Hit’ phenotype. Our works provided a resource for precision chemotherapy in the *TTN*/*OBSCN* mutant phenotypes.

## 4 DISCUSSION

In the present study, we identified the mutations of *TTN* and *OBSCN* were significantly associated with higher TMB and favourable OS. Given the significant commutation phenomenon of *TTN* and *OBSCN*, CRC patients were stratified into ‘Double‐WT’, ‘Single‐Hit’ and ‘Double‐Hit’ phenotypes. The three phenotypes presented distinct clinical and molecular characteristics as well as immune landscape, suggesting specific therapeutic and management strategies required for each phenotype. Importantly, patients with *TTN*/*OBSCN* ‘Double‐Hit’ belonged to ‘immune‐hot’ tumours and potentially better immunotherapeutic efficacy. We also identified plenty of latent chemotherapeutic agents with specific sensitivity among three phenotypes, providing a resource for precision chemotherapy in the *TTN*/*OBSCN* mutant phenotypes. Collectively, this study underlined the importance of FMGs in CRC and proposed a novel mutational classification for clinical translation, especially for predicting the responses to immunotherapy.

Both *TTN* and *OBSCN* belong to the family of giant sarcomere signalling proteins and have a role in the organization of myofibrils during assembly, which may mediate interactions between the sarcoplasmic reticulum and myofibrils.^25^, ^26^ Previous study has demonstrated that *TTN* and *OBSCN* were driver genes of CRC, but their clinical significance has not been elaborated.^27^ In our research, *TTN* and *OBSCN* mutations were common genomic events in both the United States and China. We found mutations in *TTN* and *OBSCN* indicated a higher TMB and were independent protective factors for OS. Intriguingly, possibly because *TTN* and *OBSCN* belonged to the same functional family, their mutations presented a significant commutation phenomenon in CRC.^28^ Based on the status of *TTN* and *OBSCN*, patients were divided into ‘Double‐WT’, ‘Single‐Hit’ and ‘Double‐Hit’ phenotypes. The three phenotypes accounted for roughly comparable proportions in the United States and China. The novel classification system was an independent prognostic factor. As mutations in *TTN* and *OBSCN* decline, patients were more prone to have a poor prognosis and malignant events (including lymphatic or distant metastasis and MSS). Moreover, the ‘Double‐Hit’ phenotype displayed the highest TMB compared to the other two phenotypes. TMB represents the accumulation of somatic mutations in tumours, and high‐TMB can increase the production of mutation‐derived neoantigens and enhance tumour immunogenicity, which might induce the proliferation and activation of cytotoxic T lymphocyte.^29^ Therefore, we suggested that the ‘Double‐Hit’ phenotype might have better immune response.

Actually, the ‘Double‐Hit’ phenotype enriched plenty of immune‐related pathways and presented the highest abundance of immune cells infiltration, indicating the ‘immune‐hot’ subtype. Previous research has reported the ‘immune‐hot’ tumours are more sensitive to immunotherapy.^30^ Beyond that, for some popular immunotherapy biomarkers in CRC, including TMB, MSI, ICPs expression and *POLE* mutations, we found their distribution in the ‘Double‐Hit’ phenotype were more conducive to obtaining an effective immunotherapy response. SubMap and TIDE analysis further validated this conclusion from the perspective of bioinformatics. Moreover, by using the TIDE algorithm, the *TTN*/*OBSCN* mutant phenotypes had better performance in identifying patients who responded to immunotherapy compared with *PD‐L1*, *PD‐1*, *CTLA‐4*, TMB and microsatellite status.

Moreover, the three phenotypes presented distinct molecular characteristics. Each phenotype exhibited a high mutation rate of *TP53*, particularly the ‘Double‐WT’ and ‘Single‐Hit’ phenotypes in the United States. Mutations lead to loss of p53 protein function, which is associated with tumour progression, high recurrence and high mortality.^20^ Tumour suppressor *SMAD4* is an essential modulator of the transforming growth factor (TGF)‐beta pathway controlling proliferation. Mutations in *SMAD4* indicate resistance to oxaliplatin‐based chemotherapy,^21^ suggesting that the ‘Double‐Hit’ phenotype with the most *SMAD4* mutations might be the least sensitive to oxaliplatin than the other two phenotypes. *BRAF* and *PIK3CA* are key driven genes in CRC, and their mutations are significantly associated with resistance to anti‐EGFR therapy such as cetuximab and panitumumab.^22^, ^23^ Obviously, the mutation rate of *BRAF* and *PIK3CA* in the ‘Double‐Hit’ phenotype was 3‐9 times higher than that in the other two phenotypes. These results suggested that patients with the ‘Double‐Hit’ phenotype may benefit less from first‐line chemotherapy for CRC such as oxaliplatin, cetuximab and panitumumab. Subsequently, as a supplement, we identified plenty of potential antitumour drugs with specific sensitivity to each phenotype, and most drugs were more sensitive to the ‘Double‐Hit’ phenotype. These results offered many opportunities for potential targeted therapies in the ‘Double‐Hit’ phenotype. Therefore, after first‐line chemotherapy fails, patients with the ‘Double‐Hit’ phenotype have many other potential chemotherapeutic options other than immunotherapy alone, because combination therapy may achieve better efficacy.

The present study also had some limitations. First, further in vivo and in vitro studies are needed to verify these findings. Second, multiple bioinformatics algorithms were utilized to evaluate the immunotherapy response of patients with CRC rather than conducting large‐scale immunotherapy clinical trials.

In conclusion, we defined a novel classification based on the mutation status of *TTN* and *OBSCN*. The three *TTN*/*OBSCN* mutant phenotypes demonstrate significantly distinct prognosis, malignant events propensity, molecular characteristics and immune landscape. The ‘Double‐Hit’ phenotype suggests ‘immune‐hot’ tumours and potentially better immunotherapeutic efficacy. The identified antitumour drugs are a resource for precision chemotherapy in the *TTN*/*OBSCN* mutant phenotypes. Overall, this study revealed CRC patients with TTN/OBSCN ‘Double‐Hit’ was significantly associated favourable prognosis, ‘immune‐hot’ subtype and potentially better immunotherapeutic efficacy, which may serve as a powerful indicator to further optimize prognostic management and immunotherapies for CRC.

## CONFLICT OF INTEREST

The authors declare that they have no competing interests.

## AUTHOR CONTRIBUTIONS

**Zaoqu Liu:** Conceptualization (lead); Data curation (lead); Formal analysis (lead); Investigation (lead); Methodology (lead); Project administration (lead); Resources (lead); Software (lead); Supervision (lead); Validation (lead); Visualization (lead); Writing‐original draft (lead); Writing‐review & editing (lead). **Libo Wang:** Conceptualization (supporting); Formal analysis (supporting); Investigation (equal); Software (equal); Supervision (equal); Validation (equal); Visualization (equal); Writing‐review & editing (equal). **Chunguang Guo:** Formal analysis (equal); Investigation (supporting); Supervision (supporting); Validation (supporting); Writing‐review & editing (supporting). **Long Liu:** Formal analysis (equal); Investigation (equal); Methodology (equal); Project administration (equal); Resources (equal); Software (equal); Supervision (equal); Validation (supporting); Writing‐review & editing (supporting). **Dechao Jiao:** Conceptualization (equal); Funding acquisition (lead); Investigation (equal); Methodology (equal); Software (equal); Supervision (equal); Writing‐original draft (equal); Writing‐review & editing (equal). **Zhenqiang Sun:** Conceptualization (equal); Data curation (equal); Funding acquisition (supporting); Visualization (equal); Writing‐original draft (supporting); Writing‐review & editing (supporting). **Kunpeng Wu:** Data curation (supporting); Formal analysis (supporting); Investigation (supporting); Methodology (supporting); Validation (supporting); Visualization (supporting); Writing‐review & editing (supporting). **Yanan Zhao:** Writing‐review & editing (equal). **Xinwei Han:** Conceptualization (lead); Data curation (equal); Formal analysis (equal); Funding acquisition (lead); Investigation (lead); Project administration (lead); Resources (lead); Validation (supporting); Writing‐original draft (lead); Writing‐review & editing (lead).

## Supporting information

Fig S1

Fig S2

## Data Availability

Public data used in this work can be acquired from TCGA (http://portal.gdc.cancer.gov/) and ICGC (http://dcc.icgc.org/) data sets.
