# Supplementary figures and images for: TTN/OBSCN ‘Double‐Hit’ predicts favourable prognosis, ‘immune‐hot’ subtype and potentially better immunotherapeutic efficacy in colorectal cancer
Source: J Cell Mol Med. 2021 Feb 23;25(7):3239–51. doi: 10.1111/jcmm.16393 (PMC8034451; doi:10.1111/jcmm.16393)

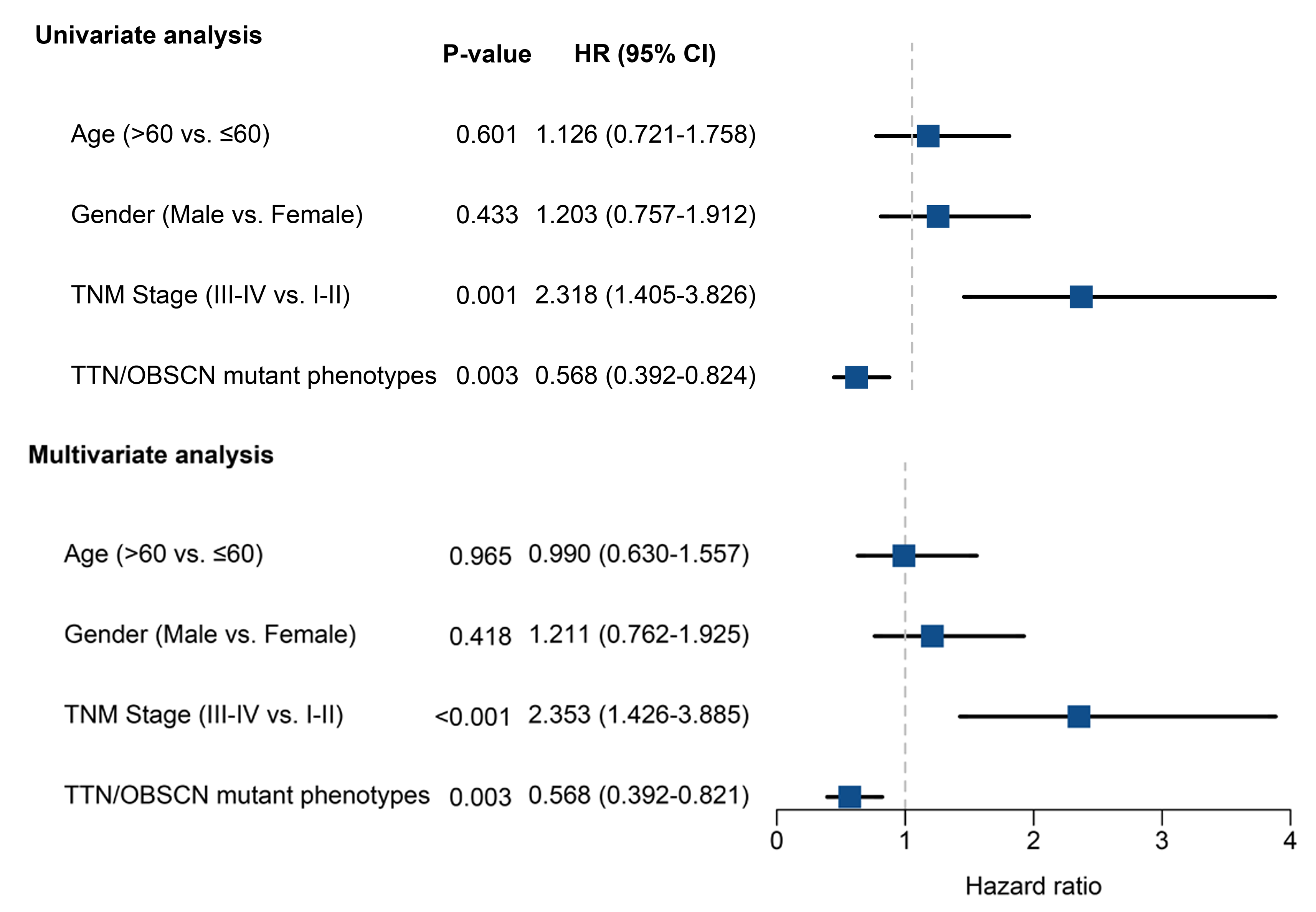

Supplement: Supplementary file 1 — Fig S1 [file JCMM-25-3239-s002.tiff]

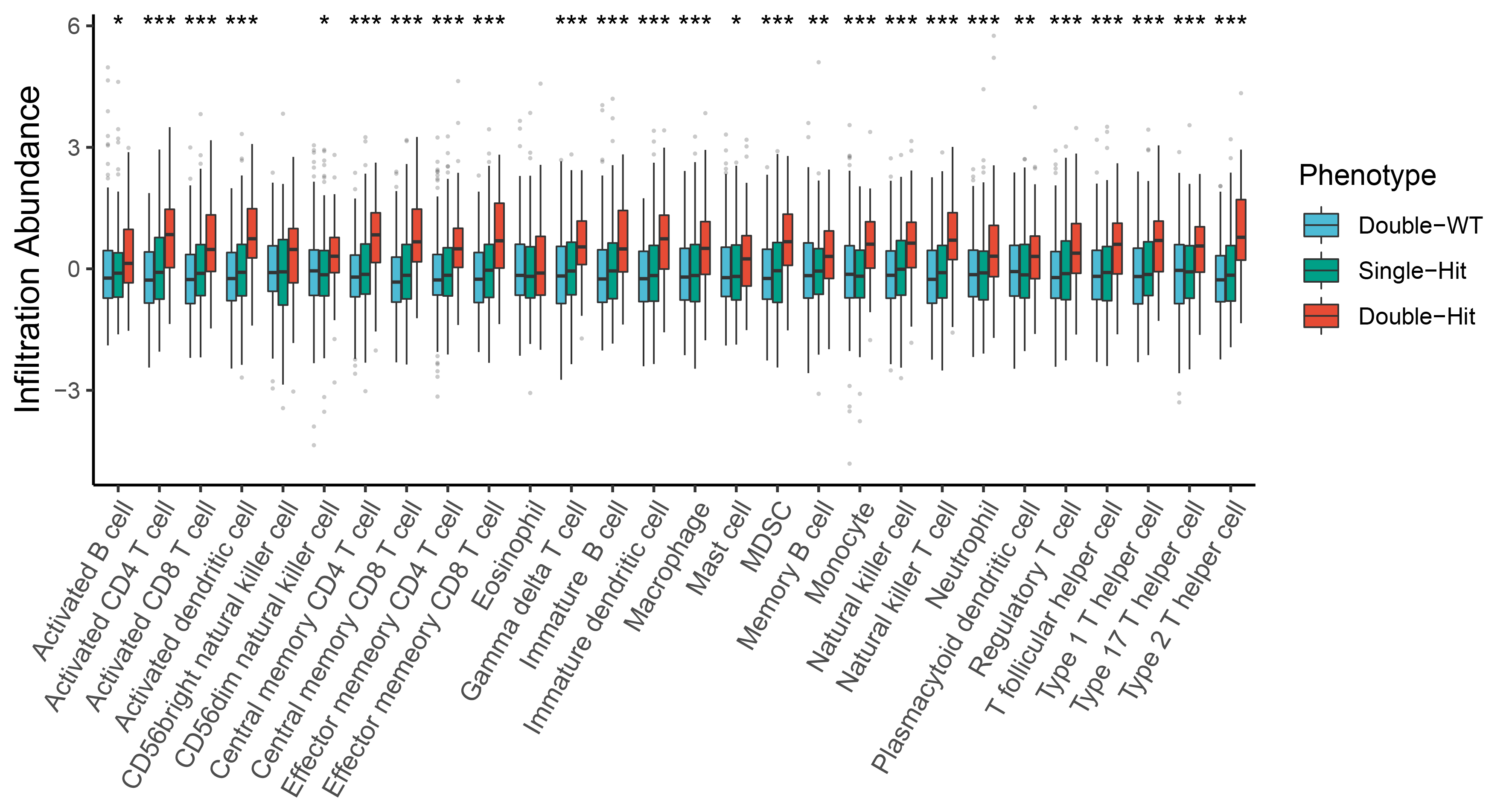

Supplement: Supplementary file 2 — Fig S2 [file JCMM-25-3239-s001.tiff]
